# Green chemistry strategies in pulping and biomass valorization: toward a circular bioeconomy

**DOI:** 10.3389/fchem.2025.1724324

**Published:** 2025-12-02

**Authors:** Neha Sharma, Priyanka Basera

**Affiliations:** 1 IFM Institute of Frontiers Material, Deakin University, Geelong, VIC, Australia; 2 Environmental and Industrial Biotechnology Division (EIBD), The Energy and Resources Institute (TERI), New Delhi, India

**Keywords:** circular bioeconomy, efficient technologies, green chemistry, organosolvent process, sustainable strategies

## Abstract

The transition toward a circular bioeconomy demands innovative, sustainable, and efficient technologies for biomass valorization and pulping. Green chemistry strategies, particularly organosolvent pulping pretreatment, are emerging as pivotal solutions to unlock the full potential of lignocellulosic feedstocks. Organosolvent processes employ environmentally benign solvents to selectively fractionate biomass components, enabling the recovery of high-purity cellulose, hemicelluloses, and lignin with minimal environmental footprint. These technologies advance the principles of green chemistry by minimizing hazardous reagents, reducing energy consumption, and promoting waste valorization. Recent developments demonstrate their capacity not only to improve pulping efficiency but also to produce value-added chemicals, biomaterials, and biofuels, thereby closing resource loops and reducing reliance on fossil-based systems. This review uniquely integrates advances in organosolvent pulping pretreatment within the framework of green chemistry and circular bioeconomy. This work systematically compares multiple green solvent systems including ionic liquids, deep eutectic solvents, and bio-derived organosolvent methods alongside catalytic, biocatalytic, and process intensification techniques. It also synthesizes recent industrial case studies, bridging the gap between laboratory research and pilot-to-commercial scale deployment. By highlighting the synergistic role of these technologies in achieving high-purity biomass fractionation with minimal environmental footprint, the review provides actionable insights for researchers, policymakers, and industry stakeholders aiming to accelerate the transition to a regenerative, circular bioeconomy.

## 1 Introduction

### 1.1 Overview of the pulp and paper industry and biomass utilization

**FIGURE 1:**
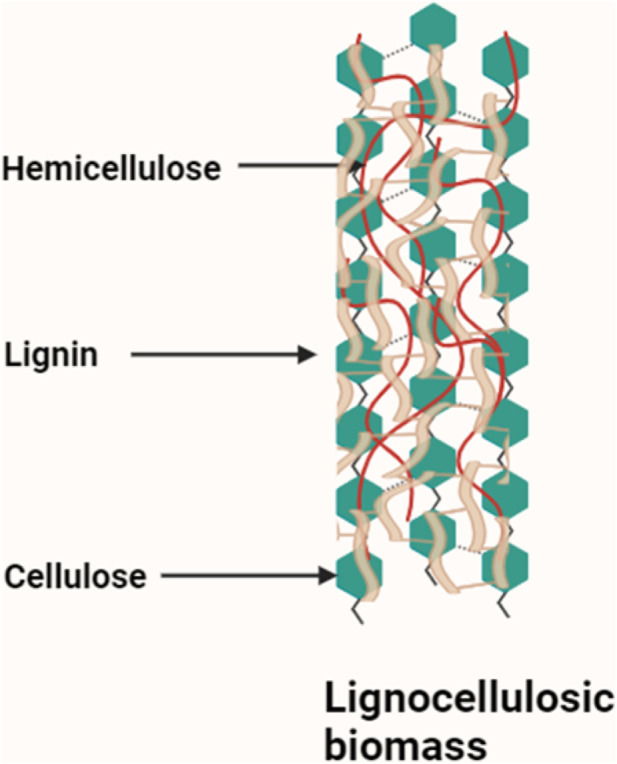
Schematic of lignocellulosic biomass structure.

The pulp and paper industry are one of the most resource-intensive sectors globally, with significant environmental and economic footprints. It plays a vital role in the global economy, producing paper, packaging materials, and textiles (Branco et al., 2019; Stoklosa and Hodge, 2014). This industry primarily relies on lignocellulosic biomass-wood, agricultural residues, and other plant-based materials such as raw feedstock (Amândio et al., 2022). The process of pulping involves separating cellulose fibers from lignin and hemicellulose to produce paper-grade pulp (Figure 1). Similarly, biomass conversion technologies aim to transform plant-derived materials into fuels, chemicals, and materials, positioning biomass as a key driver of the emerging bioeconomy (Nocito et al., 2025).

Beyond the paper industry, lignocellulosic biomass is being recognized for its potential to replace fossil-based raw materials in a variety of sectors including energy, construction, pharmaceuticals, and packaging (Baruah et al., 2018; Canilha et al., 2013; Chen and Fu, 2016; Fatma et al., 2018; Seidl and Goulart, 2016; Souza et al., 2019; Zabed et al., 2016; Zheng et al., 2014). Agricultural residues (such as wheat straw, rice husk, and sugarcane bagasse) and forestry by-products are now being explored as sustainable feedstocks for biorefineries that integrate multiple value chains.

### 1.2 Challenges of conventional pulping and conversion

Despite its industrial significance, conventional pulping and biomass conversion technologies pose several sustainability challenges *viz*. High energy and water consumption, traditional kraft and sulfite pulping processes require high temperatures (over 150 °C) and pressures, contributing to significant energy consumption and water usage. Chemical pollution and waste, the use of sulphur-based chemicals, chlorine, and strong alkalis results in the generation of toxic effluents (e.g., black liquor), which, if not treated properly, can harm aquatic ecosystems (Haile et al., 2021; Song K. et al., 2019). Further, air emissions, the combustion of fossil fuels during pulp processing leads to the emission of greenhouse gases (GHGs), volatile organic compounds (VOCs), and sulphur oxides (SOx), contributing to climate change and air pollution (Hao and Mi, 2016; Rio et al., 2022; van Ewijk et al., 2021). Moreover, in lignin underutilization, lignin, which accounts for up to 30% of plant biomass, is often burned for energy recovery instead of being vaporized into high-value products such as resins, adhesives, or carbon fibers (Basera et al., 2024). Therefore, the increasing costs of energy, regulatory pressures on pollution control, and growing demand for sustainability place economic stress on traditional pulp mills. Collectively, these challenges underscore the need for innovative, environmentally friendly, and economically viable alternatives for pulping and biomass valorization.

### 1.3 Green chemistry for a circular bioeconomy

Green chemistry, also referred to as sustainable chemistry, which is the design of chemical products and processes that reduce or eliminate the use or generation of hazardous substances (Amato and Korhonen, 2021; Ncube et al., 2023). Coined in the 1990s, green chemistry is governed by some foundational principles proposed by Paul Anastas and John Warner (Marco et al., 2019). These principles include waste prevention such as, design for energy efficiency, use of renewable feedstocks, safer solvents and reaction conditions, catalysis over stoichiometric reagents and design for degradation and non-toxicity (Oliveira et al., 2024). Further, applying these principles to biomass processing involves creating technologies that are non-toxic, energy-efficient, atom-economical, and capable of utilizing biomass components completely without generating significant waste or pollution.

The circular bioeconomy combines the principles of a circular economy which emphasizes resource efficiency, reuse, recycling, and minimal waste with the use of biological resources as feedstocks. In this context, the valorization of agricultural residues, forest waste, and industrial biomass is essential. A circular bioeconomy not only reduces dependence on fossil resources but also adds economic value to waste streams, mitigates climate change, and supports rural and regional development (Guarieiro et al., 2022).

This review aims to provide a comprehensive overview of how green chemistry strategies are revolutionizing pulping and biomass conversion processes, with a specific focus on their contributions to building a circular bioeconomy. By synthesizing the latest scientific developments, industrial case studies, and emerging technologies, the review seeks to highlight both progress and challenges in the field. The review serves multiple objectives: firstly, to analyze the limitations of conventional pulping and conversion technologies, secondly, to explore green alternatives such as ionic liquids, deep eutectic solvents, enzymatic processes, and catalytic systems. Further, to assess the integration of these strategies into biorefineries aimed at zero-waste production. Lastly, to identify gaps in current research and propose future directions for sustainable industrial practice.

## 2 Environment and technical limits of pulping

Despite being foundational to several industries especially paper, packaging, biofuels, and bio-based chemicals, these conventional pulping and biomass processing technologies are fraught with environmental, operational, and economic drawbacks (Gavrilescu et al., 2012). These drawbacks undermine the long-term sustainability of such systems and emphasize the urgent need for greener, more efficient alternatives.

### 2.1 Environmental footprint: toxic effluent generation

One of the most pressing environmental concerns associated with traditional pulping methods (especially Kraft and sulfite processes) is the generation of toxic effluents. These processes involve harsh chemicals such as sodium hydroxide (NaOH), sodium sulfide (Na_2_S), sulphurous acid, and chlorine-containing compounds, which break down lignin and separate cellulose fibers from biomass (Chrvalová et al., 2025; Li et al., 2023; Mboowa, 2024). Black liquor, a by-product of Kraft pulping, contains a complex mixture of lignin fragments, carbohydrates, sulfides, and heavy metals. Without adequate treatment, its discharge into water bodies can severely affect aquatic ecosystems by depleting oxygen and introducing carcinogenic compounds (Mandeep et al., 2019).

**FIGURE 2:**
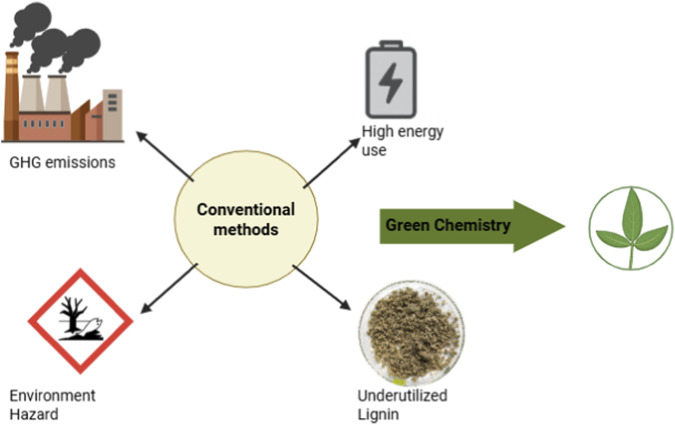
Environmental and technical constrains of conventional pulping (Schematic showing effluent discharge, high energy use, GHG emissions, and lignin underutilization compared to a green chemistry pathway).

Bleaching processes, particularly those using elemental chlorine or chlorine dioxide, produce organochlorine compounds, including dioxins and furans, which are persistent, bioaccumulative, and highly toxic to both humans and wildlife (Jaiswal et al., 2021; Puzyn and Mostrag 2012; Sharma and Singh, 2021; Singh and Chandra 2019). Many developing nations still lack advanced wastewater treatment infrastructure, leading to direct or indirect release of these effluents into the environment (Figure 2).

#### 2.1.1 Greenhouse gas emissions

Conventional biomass processing is highly energy-intensive, often relying on fossil fuels to maintain the high temperatures and pressures required for pulping and hydrolysis. This leads to substantial CO_2_, SOx, and NOx emissions from boilers, reactors, and transportation. In Kraft pulping, fossil-based fuels and black liquor are burned for energy and recovery, but this combustion also emits large amounts of GHGs, particularly if the process lacks modern emission control technologies (Forster et al., 2023; Miner, and Upton 2002; Rajan et al., 2024; Sun et al., 2018; Tomberlin et al., 2020). And in biomass conversion facilities using thermochemical routes (like pyrolysis or gasification), methane and carbon monoxide may also be released if combustion or cracking is incomplete (Jaiswal et al., 2021). These emissions contribute not only to climate change but also to local air quality issues, such as smog formation and acid rain, affecting public health and biodiversity.

### 2.2 Inefficiencies in resource utilization: underutilization of lignin

Lignin, the second most abundant polymer in plant biomass, is a highly aromatic compound with potential applications in adhesives, carbon fibers, resins, and value-added chemicals. However, in most traditional pulping operations, lignin is treated as waste or fuel and is burned for energy recovery (Basera et al., 2024). Only a small fraction (<5%) of industrially extracted lignin is valorized into specialty products. Further, its complex, heterogeneous structure post-extraction (due to harsh chemical degradation) makes it difficult to purify or convert efficiently (Brenes et al., 2022). This represents a significant loss of chemical and economic value, especially in the context of a circular bioeconomy where all biomass components should be utilized optimally.

#### 2.2.1 Biomass waste generation

Traditional processes are optimized for cellulose recovery but often ignore or discard the non-cellulosic fractions such as hemicellulose, extractives, and pectin-rich materials. This leads to incomplete utilization of raw biomass, reducing process efficiency and increasing feedstock costs and solid waste generation, which must be managed or disposed of, increasing operational burden (Li et al., 2023). Additionally, agricultural residues like straw, husks, and bagasse are often open-burned or landfilled due to lack of efficient valorization methods, contributing further to environmental pollution and missed opportunities for renewable feedstock utilization (Amode and Jeetah, 2020).

### 2.3 Economic and operational barriers: high energy and chemical inputs

The energy demand of traditional pulping and biomass conversion is significant. Processes typically require, High temperatures (140 °C–180 °C) for chemical reactions (e.g., lignin breakdown). Elevated pressures, necessitating specialized reactors and safety systems. And Large volumes of chemicals, many of which are toxic, corrosive, or expensive (Lopez-Beceiro et al., 2021; Abolore et al., 2025). These factors translate into high operational costs, especially as energy prices rise and environmental regulations tighten. Moreover, the recovery and recycling of spent chemicals (e.g., through recovery boilers or chemical loops) require additional infrastructure and maintenance.

#### 2.3.1 Scalability concerns

Although the pulp and biomass industries are established at large scales, innovations and transitions toward more sustainable systems face significant scaling hurdles. Such as, retrofitting existing plants to incorporate green processes (e.g., ionic liquids, enzymatic systems) can be capital-intensive and technically challenging. Further, new green technologies, such as biocatalysis or deep eutectic solvents (DES)-based pulping, often demonstrate excellent lab-scale results but encounter scalability, stability, and reproducibility issues when tested in pilot or industrial settings (Chrvalová et al., 2025; Da Costa Lopes et al., 2020; Lim et al., 2019; Smink et al., 2020). Moreover, the variability of biomass feedstocks—in terms of moisture content, lignin composition, and seasonal availability—further complicates process standardization. Together, these barriers limit the commercial viability of more sustainable approaches unless they are optimized for cost-efficiency, ease of integration, and regulatory compliance.

## 3 Green chemistry for sustainable biomass conversion

The integration of green chemistry into biomass conversion represents a foundational shift in how materials are processed and utilized. Rather than focusing solely on end-of-pipe solutions for pollution control, green chemistry emphasizes preventive strategies that minimize environmental and human health impacts at the source (Slootweg 2024). When applied to pulping and biomass valorization, green chemistry not only reduces ecological footprints but also enhances process efficiency, product quality, and economic viability (Maqsood et al., 2025; Xie et al., 2023).

In this context, green chemistry serves as a cornerstone of the circular bioeconomy, ensuring that biomass utilization aligns with resource conservation and waste minimization goals. The core principles most relevant to sustainable biomass processing include atom economy, which emphasizes the maximization of material incorporation into final products; the use of safer solvents and auxiliaries, prioritizing low-toxicity, biodegradable, and recyclable media such as ionic liquids (ILs) and deep eutectic solvents (DES); catalysis, which enables reactions under milder conditions with higher selectivity; and energy efficiency, achieved through emerging techniques like microwave- and ultrasound-assisted processes (Smink et al., 2020; Withouck et al., 2023).

### 3.1 Atom economy

Atom economy is a central principle of green chemistry that emphasizes the efficient incorporation of all reactant atoms into the final desired product, thereby minimizing waste and improving resource utilization (Li and Trost, 2008). A high atom economy indicates a process that maximizes product yield while reducing the formation of unwanted by-products or residues. In biomass processing, fragmentation of lignocellulosic structures should be designed to maximize the yield of target molecules (e.g., sugars, aromatic monomers) while minimizing by-products (Chiang et al., 2024). And reductive catalytic fractionation (RCF) exemplifies high atom economy by selectively depolymerizing lignin into usable monomers without unnecessary degradation of cellulose (Arts et al., 2023; Renders et al., 2019).

### 3.2 Safer solvents and auxiliaries

Conventional pulping and biomass processing methods frequently rely on hazardous solvents and reagents, such as sulfur-based compounds (e.g., sodium sulfide in kraft pulping) and chlorine-based bleaching agents, which pose significant risks to both the environment and human health. These chemicals generate toxic effluents, contribute to air and water pollution, and compromise the overall sustainability of industrial operations (Hubbe et al., 2019). In contrast, green chemistry promotes the replacement of such harmful substances with benign, biodegradable, and recyclable alternatives, thereby shifting focus from pollution control to pollution prevention (Anastas and Eghbali, 2010).

Among the most promising innovations are ionic liquids (ILs) and deep eutectic solvents (DES), which have emerged as effective green solvents for biomass dissolution and fractionation. ILs, comprising organic cations and various anions—exhibit negligible vapor pressure, high thermal stability, and tunable solvation properties, allowing selective dissolution of cellulose, hemicellulose, and lignin under mild conditions (Binnemans and Jones, 2023; Smink et al., 2020). Similarly, DES, formed by the combination of a hydrogen bond and are cost-effective, biodegradable, and easily synthesized from renewable precursors such as choline chloride, glycerol, and organic acids (Chrvalová et al., 2025; Guazzelli et al., 2023). Their unique hydrogen-bonding networks facilitate efficient biomass pretreatment, enhancing enzymatic hydrolysis and reducing lignin recalcitrance without generating toxic residues.

Ideally, green solvents used in pulping and biorefinery applications should be non-toxic, non-flammable, biodegradable, and derived from renewable resources, thereby satisfying the dual imperatives of environmental safety and circularity.

### 3.3 Catalysis

Catalysis lies at the heart of green chemistry, serving as a key strategy to minimize waste, reduce energy demand, and improve selectivity in chemical transformations (Kaur et al., 2024). Unlike stoichiometric processes that consume large quantities of reagents and generate by-products, catalytic systems enable multiple reaction cycles with minimal input, thereby enhancing atom economy and overall process sustainability. In biomass conversion, catalysis plays a pivotal role in unlocking the chemical potential of lignocellulosic feedstocks. Heterogeneous and homogeneous metal catalysts—including ruthenium (Ru), palladium (Pd), nickel (Ni), and copper (Cu)—are widely employed in hydrogenolysis, hydrodeoxygenation, and reductive catalytic fractionation (RCF) to depolymerize lignin into high-value aromatic monomers and phenolic intermediates (Akhtar et al., 2025; Anderson et al., 2018; Bosch et al., 2015; Oh et al., 2022). Further, biocatalysts (enzymes) such as cellulases, xylanases, and laccases offer substrate specificity under mild conditions, enabling environmentally friendly and highly efficient biomass breakdown.

### 3.4 Energy efficiency

Minimizing energy consumption in chemical processes is essential for sustainability. In the context of green chemistry, the objective is to maximize reaction efficiency while operating under ambient or near-ambient temperature and pressure, thereby reducing both the carbon footprint and overall operational costs (Singh et al., 2010). Energy-efficient biomass processing technologies are designed to optimize heat and mass transfer, minimize energy dissipation, and exploit alternative energy sources that offer superior control and selectivity. Among such innovations, microwave-assisted and ultrasound-assisted pretreatments have gained prominence for their ability to enhance reaction kinetics and solvent penetration without the excessive thermal gradients typical of conventional heating (Lozano et al., 2024). Similarly, ultrasound-assisted processes leverage acoustic cavitation to disrupt biomass structure, increasing surface accessibility for enzymatic or catalytic conversion (Göncü, et al., 2025). In parallel, supercritical CO_2_ extraction exemplifies a clean, energy-efficient separation technology that utilizes tunable pressure and temperature conditions to dissolve and fractionate bioactive compounds and lignocellulosic constituents without generating hazardous waste. Together, they form the blueprint for developing next-generation biomass valorization processes that are environmentally benign, economically feasible, and aligned with circular bioeconomy goals.

#### 3.4.1 Translating concepts of green chemistry into practice

The practical application of green chemistry principles in biomass processing is increasingly visible across multiple domains, including solvent design, process engineering, and waste valorization. These real-world implementations mark a departure from traditional “end-of-life” waste treatment to front-end design improvements.

#### 3.4.2 Application in solvent design

Solvent systems are a critical component in pulping and biomass pretreatment, responsible for dissolving lignin and hemicellulose while preserving cellulose. Custom-designed ionic liquids with task-specific cations and anions are being engineered to selectively extract certain biomass components. For example, imidazolium-based ILs can effectively solubilize cellulose while leaving lignin intact, or *vice versa*, depending on the anion used (e.g., acetate, chloride) (Du and Qian, 2011; Singh et al., 2018; Verdía Barbará et al., 2025). Further, natural deep eutectic solvents (NADES), composed of bio-based components such as choline chloride, glycerol, and lactic acid, offer biodegradable, non-toxic alternatives to petroleum-based solvents (Hikmawanti et al., 2021; Satija et al., 2024; Usmani et al., 2023). These designer solvents not only improve efficiency and selectivity, but also enable solvent recycling, critical for industrial scalability.

#### 3.4.3 Process engineering innovations

Modern biomass processing integrates green chemistry through smarter, cleaner engineering solutions through enzymatic pulping which avoids harsh chemicals by using laccases, peroxidases, and xylanases to degrade lignin and hemicellulose (Ali et al., 2024; Sah et al., 2025). These enzymes work under aqueous, mild conditions, reducing energy and chemical inputs. Furthermore, integration of Reductive Catalytic Fractionation (RCF) which combines solvent extraction and catalytic conversion in a single step to selectively cleave ether bonds in lignin and produce valuable phenolic monomers (Renders et al., 2019). Incorporation of advanced technologies (Supercritical and Microwave-Assisted Systems) allow faster reaction times, reduced solvent volumes, and better selectivity without extreme operating conditions. These engineering innovations reflect a shift toward closed-loop systems and low-waste operations, which are essential for circularity.

#### 3.4.4 Waste valorization

Instead of treating lignin, hemicellulose, or residual biomass as waste, green chemistry promotes their transformation into value-added products (Jiju et al., 2025). Such as, lignin, once considered an industrial by-product, can now be upgraded into bio-based adhesives, aromatic monomers, and activated carbon (Den et al., 2018; Rath et al., 2024). Further, hemicellulose-derived sugars (e.g., xylose, arabinose) serve as feedstocks for xylitol, furfural, and bio-based polymers (Polipalli et al., 2025). And residual biomass can be converted into biochar, biocomposites, or platform chemicals, ensuring that every fraction of the biomass feedstock is utilized. By turning waste into resources, these strategies align with both green chemistry and circular bioeconomy principles, closing the loop and improving resource efficiency.

## 4 Green solvent systems for pulping and biomass fractionation

Traditional pulping and biomass fractionation methods rely heavily on toxic and non-renewable solvents, such as sodium hydroxide, sulfites, and chlorine-based chemicals. These solvents, while effective, pose significant challenges in terms of waste management, corrosion, toxicity, and environmental persistence. Green chemistry offers innovative alternatives in the form of green solvent systems—especially ionic liquids (ILs), deep eutectic solvents (DES), and organosolv systems—that enable efficient biomass fractionation while aligning with sustainability goals (Binnemans, and Jones 2023; Singh et al., 2018; Smink et al., 2020). This section examines the chemical principles, operational benefits, and current limitations of these green solvents and their roles in biomass processing.

### 4.1 Ionic liquids (ILs) in biomass dissolution

Ionic liquids are salts with melting points below 100 °C, typically composed of bulky organic cations (e.g., imidazolium, pyridinium, cholinium) and diverse anions (e.g., chloride, acetate, phosphates) (Mital et al., 2021). Their key physicochemical properties—non-volatility, thermal stability, low vapor pressure, high solvating ability, and tunability—make them particularly suited for biomass dissolution. Imidazolium-based ILs, such as 1-ethyl-3-methylimidazolium acetate ([EMIM][OAc]), have demonstrated exceptional ability to dissolve cellulose, disrupt hydrogen bonding in lignocellulosic biomass, and facilitate enzymatic saccharification (Salas et al., 2023). Further, ILs can selectively solubilize lignin, hemicellulose, or cellulose, depending on their structure and acidity/basicity, enabling fractionated recovery of biomass components. This technique as various advantages and limitation. Advantages include highly selective dissolution of biomass fractions and enable low-temperature processing. Moreover, facilitate recyclability and reuse and enhance enzyme accessibility by modifying biomass crystallinity. In addition to this, limitation are high cost and synthetic complexity of ILs limit large-scale applications. Some ILs exhibit toxicity toward microbes and enzymes, affecting downstream bioconversion (Grewal et al., 2022). Further, difficulties in complete solvent recovery and purification and corrosiveness in certain formulations (especially chloride-based Ils). Despite these drawbacks, ongoing research focuses on low-cost, biodegradable, and task-specific ILs, particularly those based on cholinium and amino acid-derived anions, to mitigate toxicity and improve sustainability.

### 4.2 Deep eutectic solvents (DES) with biomass pretreatment potential

DES are liquid mixtures of hydrogen bond donors (HBDs) and acceptors (HBAs) that form eutectic systems with melting points significantly lower than their individual components (Nahar and Thickett, 2021). Common HBA are choline chloride (vitamin B4 derivative) and common HBDs are urea, glycerol, lactic acid, oxalic acid. The eutectic interaction disrupts biomass recalcitrance by cleaving lignin–carbohydrate complexes and altering biomass structure, enabling better enzyme access (Rajapakse et al., 2025). Choline chloride–lactic acid DES is a notable system for lignin extraction. And DES pretreatment leads to reduced cellulose crystallinity and increased hemicellulose solubilization, improving downstream saccharification. A choline chloride–glycerol DES removed up to 85% of lignin from wheat straw under mild conditions (∼100 °C), enhancing glucose yield during enzymatic hydrolysis (Tang et al., 2023). Another study reflects that in corn stover pretreatment, DES-based methods achieved comparable lignin removal efficiency to organosolv systems but with lower toxicity and simpler recovery (Cronin et al., 2020).

### 4.3 Organosolv processes

Organosolv pulping utilizes organic solvents (alcohols, acids, or ketones) often mixed with water to dissolve lignin and hemicellulose from lignocellulosic biomass. Common solvents used for the pulping are ethanol, methanol, lactic acid, acetic acid, and γ-valerolactone (Nair et al., 2023; Tofani et al., 2024). These systems often incorporate mild acid catalysts (e.g., H_2_SO_4_, oxalic acid) to enhance delignification. Organosolv processes allow selective lignin removal, yielding high-purity cellulose pulp and sulphur-free lignin suitable for high-value applications (Table 1).

**TABLE 1:** Insights of the solvents systems based on their properties, composition and application.

Solvent system	Composition and properties	Biomass application	Advantages	Limitations	Key references
Ionic liquids (ILs)	Organic cation + inorganic/organic anion; low vapor pressure, tunable polarity	Dissolution of cellulose and lignin; selective fractionation	- High dissolution efficiency- tunable for specific biomass components- recyclable	- High cost- potential toxicity- corrosive in some cases- complex recovery	Sun et al., 2009; Brandt et al., 2013
Deep eutectic solvents (DES)	HBA (e.g., choline chloride) + HBD (e.g., urea, glycerol); form eutectic mixture with low melting point	Lignin removal, cellulose decrystallization, biomass pretreatment	- Biodegradable- low toxicity- renewable components- cost-effective	- High viscosity- water sensitivity- limited recyclability	Francisco et al., 2013; Smith et al., 2014
Organosolv systems	Organic solvents (ethanol, methanol, lactic acid) with/without catalyst	Delignification, high-purity cellulose pulp production	- Sulphur-free lignin- high selectivity- scalable process	- Requires solvent recovery- flammability concerns- some solvents still fossil-derived	Zhao et al. (2009)

## 5 Catalytic and biocatalytic innovations

Recent advancements in catalysis and biocatalysis offer innovative and environmentally benign routes to valorize lignocellulosic biomass. These technologies facilitate selective fractionation, efficient conversion, and product diversification while adhering to green chemistry principles. This section explores the mechanistic basis, key catalysts, and system-level integration of catalytic and biocatalytic strategies in biomass processing.

### 5.1 Reductive catalytic fractionation (RCF)

Reductive Catalytic Fractionation (RCF) is a lignin-first biorefining strategy that combines solvolysis and catalytic hydrogenolysis under reductive conditions. The process targets the selective depolymerization of lignin while stabilizing the resulting monomers to avoid repolymerization (Renders et al., 2019). RCF solubilizes lignin from lignocellulosic biomass using an organic solvent (e.g., methanol, ethanol) in the presence of a heterogeneous catalyst (e.g., Pd/C, Ru/C, Ni-based catalysts) and a hydrogen source. Simultaneously, the cellulose-rich pulp remains in the solid phase for subsequent utilization.

**FIGURE 3:**
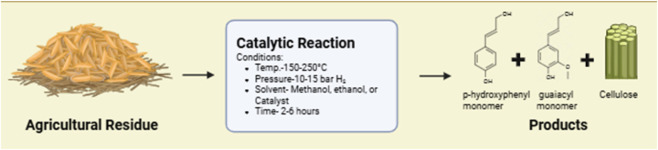
Reductive catalytic fractionation (RCF) mechanism.

RCF promotes lignin valorization at the very beginning of biomass processing, unlike traditional methods that degrade or burn lignin (Jang et al., 2022). The aromatic monomers derived can be converted into bioplastics (e.g., lignin-derived polyesters), biobased resins and adhesives, and antioxidants, surfactants, and fine chemicals. Thus, RCF aligns closely with circular bioeconomy principles by enabling closed-loop chemical recovery and value-added lignin utilization (Figure 3).

### 5.2 Biocatalysis in biomass conversion

Biocatalysis harnesses microbial enzymes to facilitate specific biochemical reactions under mild and environmentally safe conditions (Sheldon and Woodley, 2018). The key enzymes involved in lignocellulosic biomass breakdown are cellulases which hydrolyze β-1,4-glycosidic bonds of cellulose to release glucose, xylanases that break down hemicellulose (mainly xylan) into xylose and other sugars (Basera et al., 2024). Further, Laccases and Peroxidases which oxidize lignin and phenolic compounds, aiding in delignification. These enzymes offer high specificity, reduce energy inputs, and generate minimal hazardous waste compared to conventional chemical treatments.

To overcome limitations like thermal instability, low reusability, and pH sensitivity, enzyme immobilization techniques are employed. These include encapsulation (e.g., in alginate or silica gels), covalent bonding on solid supports (e.g., magnetic nanoparticles, zeolites), and cross-linking enzyme aggregates (CLEAs) (Ma et al., 2025). Immobilized enzymes exhibit enhanced thermal and operational stability, can be reused for multiple cycles and are compatible with continuous bioreactor systems (Maghraby et al., 2023).

## 6 Process intensification techniques in green pulping

Process intensification (PI) refers to strategies that enhance mass and energy transfer, shorten reaction times, and improve resource efficiency without compromising product quality (Haase et al., 2022; Li et al., 2024). In the context of pulping and biomass fractionation, PI aligns with green chemistry goals by minimizing chemical use, reducing energy demand, and lowering environmental impact. Key PI methods gaining attention include microwave-assisted pulping, ultrasound-assisted extraction, and supercritical/subcritical fluid applications.

### 6.1 Microwave-assisted pulping (MAP)

Microwave-assisted pulping utilizes electromagnetic radiation (300 MHz-300 GHz) to directly heat biomass via dipole rotation and ionic conduction. Unlike conventional heating, microwaves provide volumetric heating, leading to rapid and uniform temperature rise (Lozano et al., 2024). During microwave heating, electrical energy is transformed into thermal energy, and the rate of this conversion can be described by Equation 1 (Karmakar and Halder, 2019).

$$P=\text{Kf}\varepsilon E^{2}\left(\tan\delta \right)$$ (1)

In this expression, **tan δ** represents the *dielectric loss tangent*, **E** is the *applied electric field strength*, **ε** denotes the *absolute dielectric constant* of the material, **f** is the *microwave frequency*, **K** is a *proportionality constant*, and **P** corresponds to the *microwave power dissipated per unit volume* of the material.

The reaction kinetics occurred at faster heating rate which reduce total reaction time from hours to minutes. It promotes selective heating of water molecules in lignocellulosic biomass, enhancing cell wall rupture and solvent penetration. Moreover, improves the reaction kinetics for delignification and hydrolysis (Allende et al., 2023; Nomanbhay et al., 2017).

### 6.2 Ultrasound-assisted extraction (UAE)

Ultrasound treatment involves the application of high-frequency sound waves (20 kHz–100 kHz) to biomass slurries (Tang and Sivakumar 2014). The acoustic cavitation effect-formation, growth, and implosion of microbubbles tend to produce localized high temperatures and pressures. The overall impact on delignification was enhanced by cell wall disruption which increase the enzyme penetration with reduced chemical usage, promotes fragmentation of lignocellulosic matrices, making lignin and hemicellulose more accessible (Göncü, et al., 2025).

The enhancement in extraction yield achieved through ultrasound-assisted extraction (UAE) cannot be ascribed to a single mechanism; rather, it results from the synergistic action of multiple processes. Among these, acoustic cavitation serves as the primary driving force (Kumar et al., 2021). The acoustic pressure generated during UAE depends on the exposure time to ultrasonic waves and can be described by Equation 2 (Karmakar and Halder, 2019).

$$P_{a}=P_{A}\sin2\pi \text{ ft}$$ (2)

where **P**_**A**_ represents the maximum pressure amplitude of the ultrasonic wave, **f** is the frequency, and **t** is the duration of exposure.

### 6.3 Supercritical and subcritical fluids: green potential of CO_2_ and water as solvents

#### 6.3.1 Supercritical CO_2_ (scCO_2_)

At T > 31.1 °C and P > 73.8 bar, CO_2_ becomes a supercritical fluid, exhibiting gas-like diffusivity and liquid-like solvating power. It is non-toxic, non-flammable, and recyclable which makes it ideal for green processing (Jahnavi et al., 2025). scCO_2_ can be used as a co-solvent in organosolv pulping to enhance lignin extraction, swelling agent to increase surface area for enzymatic hydrolysis, carrier for volatile components during biomass pretreatment (Badgujar et al., 2021; Gao et al., 2010; Neata et al., 2015; Sajal, and Sutar, 2025).

Subcritical water at T = 100 °C–374 °C and P > atmospheric pressure, remaining in liquid state and acts as a green hydrolytic medium for biomass, capable of breaking down hemicellulose and lignin without added catalysts (Zhou et al., 2023). It tends to generate hydronium ions (H_3_O^+^) at elevated temperatures, offering auto-catalysis for hydrolysis reactions (Sarker et al., 2021). Multitude of advantages associated with it such as it avoids hazardous organic solvents, high reaction rates and selectivity, produces minimal secondary pollutants and allows easy recovery of valuable fractions, e.g., lignin oil, hemicellulose sugars, and cellulose pulp (Table 2).

**TABLE 2:** Comparative analysis of techniques based on their principles, benefits and output.

Technique	Principle	Benefits	Target output	Reference
Microwave-assisted pulping (MAP)	Dielectric heating via electromagnetic radiation causes rapid, uniform heating	Reduces reaction time and energy use; enhances lignin solubilization and enzyme penetration	High-purity cellulose pulp; improved lignin extraction	Camani et al., 2020; Lozano et al., 2024
Ultrasound-assisted extraction (UAE)	Cavitation effect causes cell disruption and improved mass transfer	Enhances solvent penetration, reduces chemical requirements, improves enzyme accessibility	Lignin, hemicellulose, and cellulose recovery; enzymatic digestibility	Dedhia et al., 2012; Flores et al., 2021
Supercritical CO_2_ (scCO_2_)	Above 31.1 °C and 73.8 bar; CO_2_ behaves as a tunable solvent	Green solvent; recyclable; enhances lignin solubilization; minimal solvent residue	Extracted lignin, hemicellulose-derived sugars	Badgujar et al., 2021; Sajal, and Sutar, 2025
Subcritical water (scH_2_O)	Hot-compressed water (100 °C–374 °C, high pressure) acts as a hydrolytic medium	No added chemicals; auto-catalysis; eco-friendly; selective hydrolysis	Hemicellulose sugars; partially delignified pulp	Singh et al. (2023)

## 7 Integrated biorefinery approaches

The integrated biorefinery is a cornerstone of the circular bioeconomy, aiming to utilize every component of lignocellulosic biomass such as cellulose, hemicellulose, and lignin in a synergistic and sustainable manner (Chemmangattuvalappil and Ng, 2013). Unlike conventional biomass processing, where large fractions (especially lignin) are underutilized or burned for energy, biorefineries are designed to fractionate biomass into its constituent polymers and convert them into a diverse array of value-added products, ranging from biofuels, biochemicals, polymers, to energy.

**FIGURE 4:**
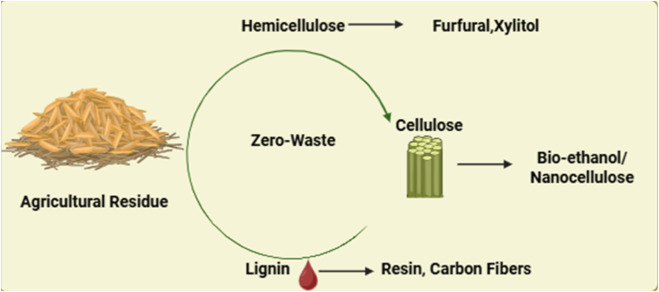
Integrated biorefinery concept.

Green chemistry principles are embedded in the design of integrated biorefineries to reduce waste, avoid toxic solvents, minimize energy consumption, and maximize product yields through selective separation and valorization. Advanced separation techniques, such as membrane filtration, organosolv pulping, ionic liquids, and deep eutectic solvents, enable selective extraction while maintaining polymer integrity (Brandt et al., 2013; Smith et al., 2014) (Figure 4).

### 7.1 Valorization pathways

A successful biorefinery does not merely process biomass, it transforms each fraction into a high-value product stream using green, economically viable routes. The valorization of lignocellulosic biomass through green chemistry approaches provides multiple sustainable pathways for value creation. Cellulose, after enzymatic hydrolysis, can be fermented to produce bioethanol, a crucial renewable fuel, or alternatively transformed into nanocellulose for applications in films, hydrogels, and biodegradable packaging materials. Green pretreatment methods such as deep eutectic solvents (DES), ionic liquids (ILs), or organosolv processes enhance enzyme accessibility while avoiding toxic residues, making the conversion more sustainable (Mital et al., 2021; Rajapakse et al., 2025). Hemicellulose, being rich in C5 sugars, can be hydrolyzed and dehydrated into furfural, an essential precursor for polymers and solvents. Green catalytic methods, particularly those using solid acid catalysts, have advanced low-temperature and energy-efficient furfural production. Additionally, hemicellulose can be converted into sugar alcohols like xylitol or organic acids such as acetic and lactic acid through fermentation or catalytic upgrading. Lignin, once treated largely as a low-value by-product or fuel, is increasingly recognized as a renewable source of aromatic compounds such as vanillin and phenol derivatives. Advanced techniques like reductive catalytic fractionation (RCF) and oxidative depolymerization selectively cleave lignin’s complex structure, yielding monomers for adhesives, resins, and fine chemicals (Renders et al., 2019; Jang et al., 2022). Beyond chemicals, lignin also holds potential for material applications, with emerging uses in carbon fibers, activated carbon, and electrode materials, further expanding its role in sustainable materials science. This holistic valorization approach supports the principles of green chemistry while advancing the goals of a circular bioeconomy.

### 7.2 Circularity and waste minimization

At the heart of the integrated biorefinery concept is the zero-waste philosophy, wherein all input biomass is transformed into products, by-products are recovered and reused, and energy is internally recycled. For instance, Borregaard Biorefinery (Norway) that converts wood into ethanol, lignin-based vanillin, and specialty cellulose—all under a low-emission, closed-loop system (Modah et al., 2015). Stora Enso Sunila Mill (Finland) that operates a lignin separation plant producing *Lineo®*, a lignin-based alternative to fossil-based binders and resins. And GranBio (Brazil) which integrates sugarcane bagasse processing into ethanol, biogas, and lignin-derived power (Björk et al., 2015). These examples underscore the potential of green integrated biorefineries to become not just sustainable processing centers, but key enablers of a circular and carbon-neutral economy.

## 8 Case studies and commercial success stories

Real-world case studies exemplify the translation of green chemistry principles into industrial practice. From lignin recovery technologies to green solvent-based pulping and enzymatic innovations, these commercial efforts demonstrate the economic viability, environmental benefits, and scalability of sustainable biomass conversion strategies.

### 8.1 LignoBoost and LignoForce technologies

LignoBoost (developed by Innventia and commercialized by Valmet) and LignoForce (developed by FPInnovations and Noram engineering) are two prominent technologies enabling industrial-scale recovery of high-purity lignin from black liquor in kraft pulp mills (Kienberger et al., 2021). LignoBoost Technology is developed in Sweden and implemented by Domtar Corporation in its Plymouth Mill, USA which involves acidification of black liquor followed by carbon dioxide precipitation to isolate lignin. The recovered lignin is low in sulphur and can be used for resins, carbon fiber, and energy applications. This process reduces sulphur emissions, improves mill energy efficiency, and offers a renewable alternative to petroleum-derived phenols. LignoForce Process is commercialized in West Fraser’s Hinton Pulp Mill, Canada that improves lignin purity by oxidizing odorous sulphur compounds before precipitation which makes it suitable for use in polymer additives, dispersants, and adhesives (Kouisni et al., 2016) (Table 3).

**TABLE 3:** Insights of case studies and success stories.

Case studies	Application area	Key features	Benefits	Limitations	Reference(s)
LignoBoost	Kraft pulp mills	CO_2_ acidification and lignin precipitation from black liquor	High-purity lignin, sulphur-free, used in polymers and fuel	Limited to kraft black liquor, needs CO_2_ handling	Kienberger et al. (2021)
LignoForce	Industrial lignin recovery	Oxidation step removes odorants before precipitation	Better odor control, high lignin purity	Pre-treatment adds complexity	Kouisni et al. (2016)
DES-based pulping	Agricultural residues (e.g., straw, bagasse)	Green solvents like ChCl–lactic acid or betaine–urea	High delignification, low temp/pressure, biodegradable	Viscosity, recovery/reuse of DES needs optimization	Francisco et al. (2012); Song B. et al. (2019)
Enzyme-assisted bleaching	Kraft and mechanical pulp bleaching	Use of xylanases, laccases, and LMS	Reduced chlorine use, lower AOX, improved brightness	Enzyme stability and cost at large scale	Bajpai (2004)

### 8.2 DES-based pulping of agricultural waste

Deep eutectic solvents (DES) have emerged as green alternatives for pulping non-woody biomass, such as wheat straw, rice husk, corn stover, and sugarcane bagasse. Their biodegradability, low toxicity, and tunability make them ideal for mild, selective fractionation of lignocellulose. Research by Francisco et al. (2012) and Song B. et al. (2019) highlighted high lignin removal (>80%) and effective hemicellulose recovery.

### 8.3 Enzyme-assisted bleaching and delignification

Enzymatic methods offer an eco-friendly solution for bleaching and delignifying pulp, reducing dependency on chlorine-based chemicals and lowering effluent toxicity (Bajpai, 2004). Novozymes and AB Enzymes have developed enzyme products (e.g., Fibrozyme®, Pulpzyme®) that are used globally in ECF (Elemental Chlorine Free) and TCF (Totally Chlorine Free) bleaching sequences.

## 9 Challenges and future perspectives

Green chemistry-based biomass valorization, though promising, faces several technical, economic, and policy barriers challenges. Despite its promise, green chemistry-based biomass valorization faces significant hurdles. Technically, solvent recovery and recyclability remain challenging as ionic liquids (ILs) and deep eutectic solvents (DES), though effective, are difficult to separate and reuse due to high viscosity and hygroscopicity. Enzyme-based processes offer selectivity but suffer from high costs, limited stability, and sensitivity to inhibitors from pretreatment, adding complexity to multi-step operations like hydrolysis and catalytic upgrading. Economically, high capital and operational costs for advanced biorefineries and expensive raw materials impede scalability. Policy gaps further exacerbate these issues, as green ventures lack incentives, subsidies, and supportive regulatory frameworks, while stringent safety and environmental approvals delay commercialization. Addressing these barriers requires innovation in task-specific solvent design, enzyme engineering for resilience and cost-efficiency, and integration of life cycle assessment (LCA) to ensure sustainability. Industrial scaling can be accelerated through modular biorefineries near feedstock sources, public-private partnerships for collaborative R&D, and capacity-building initiatives such as pilot units and training programs to bridge knowledge gaps and foster readiness (Mital et al., 2021).

## 10 Conclusion

The integration of green chemistry principles into pulping and biomass valorization offers a transformative pathway toward a sustainable and circular bioeconomy. Conventional pulping technologies, while industrially established, are limited by their environmental footprint, underutilization of lignin, and economic inefficiencies. Green alternatives such as ionic liquids, deep eutectic solvents, and organosolvent systems provide cleaner and more selective fractionation routes, while catalytic, biocatalytic, and hybrid systems enable efficient conversion of lignocellulosic polymers into value-added products. Complementary process intensification techniques, including microwave- and ultrasound-assisted methods and supercritical fluids, further enhance efficiency while minimizing chemical and energy inputs. A key novelty of this review lies in its holistic comparison of green solvent systems with catalytic and process-intensified methods, framed within the context of the circular bioeconomy. By bridging laboratory-scale innovations with industrial case studies such as LignoBoost, LignoForce, and DES-based pulping, the review underscores the growing feasibility of scaling sustainable biorefinery concepts.

Looking ahead, progress will require advancements in solvent recovery technologies, robust enzyme engineering, and integrated life cycle assessments to ensure true sustainability. Equally critical are supportive policy frameworks, public–private partnerships, and modular biorefinery models tailored to regional biomass availability. In essence, green chemistry not only mitigates the drawbacks of conventional pulping but also unlocks new economic opportunities through full biomass valorization. Its successful integration into industrial practice will be instrumental in accelerating the transition from a fossil-dependent paradigm to a regenerative, circular bioeconomy.
